# Cardiac surgical aspects of Down syndrome

**DOI:** 10.1186/1749-8090-8-S1-P96

**Published:** 2013-09-11

**Authors:** I Mokryk, O Koval, A Novack, O Kartashova, O Muzychin, O Bordyugova, V Volodyn, A Nechyporchuk, R Klymanskiy, V Konov

**Affiliations:** 1 Department of Cardio Surgery, Cardiology and Rehabilitation for Children, Government Institution “Institute of Urgent and Recovery Surgery named after V.K. Gusak National Academy of Medical Science of Ukraine”, Donetsk, Ukraine; 2 Pediatric Subdepartment of Internship and Postgraduate Education Faculty of Donetsk National Medical University n.a. M. Gorkiy, Donetsk, Ukraine

## Background

The number of children with Down syndrome (DS) in general population reaches 4,8-7,0%. More than 45,0% of them have different forms of congenital heart diseases (CHD), which is the main death cause in this patient group under two years of age. The purpose of this study was to analyze details of perioperative period in children with DS undergoing CHD repair.

## Methods

Between 2009 and 2012, 34 consecutive patients with DS and CHD were admitted for medical or surgical treatment. There were 19 (56,0%) boys and 15 (44,0%) girls aged from 4 months to 18 years (20 month ± 2 month). Most common CHD was AVSD, witch had 16 (47.1%).

## Results

82,5% (n=28) had hard combined CHD, 82,4% (n=28) - high pulmonary hypertension, all patients - heart failure. In 20,6% (n=6) patients heart catheterization was performed. 6 (20,6%) kids received only medical treatment: 2 (5,8%) - inoperable, 3 (8,8%) medical preparation for surgery due to PH, 1 (2,9%) - no need of surgery. 28 (82,4%) patients were operated on. 26 (92,8%) were open heart cases. 57,6% (n=15) underwent septal defects (ASD, VSD) plasty at median age of 12 ± 3 months. In 42,3% (n=11) AVSD was corrected at median age of 6±1 month. In 7 patients (23,6%) postoperative period was complicated by: DIC syndrome (57,1%), sepsis (28,6%), chylothorax (14,3%). 30-day in hospital mortality occurred in 3 (10,7%) cases: 1 (14,3%) – due to intractable pulmonary hypertensive crisis; 1 (14,3%) – sepsis; 1 (14,3%) – sepsis associated with DIC syndrome. Neither late death nor any residual defects requiring reoperation were detected at follow up.

## Conclusions

Surgical correction of CHD in children with DS can be accomplished with acceptable morbidity and mortality rates. Improvement of medical results is possible with special emphasis on preoperative diagnosis and treatment of complications of CHD (PH) and associated pathology (hematologic disorders, infections and immunity status disorders).

